# Evaluation of the Relationship among Biogenic Amines, Nitrite and Microbial Diversity in Fermented Mustard

**DOI:** 10.3390/molecules26206173

**Published:** 2021-10-13

**Authors:** Yangyang Yu, Lu Li, Yujuan Xu, Kejing An, Qiao Shi, Yuanshan Yu, Zhenlin Xu

**Affiliations:** 1 Guangdong Provincial Key Laboratory of Food Quality and Safety, College of Food Science, South China Agricultural University, Guangzhou 510610, China; hnyuyang@163.com; 2 Sericultural & Agri-Food Research Institute, Guangdong Academy of Agricultural Sciences/Key Laboratory of Functional Foods, Ministry of Agriculture and Rural Affairs/Guangdong Key Laboratory of Agricultural Products Processing, Guangzhou 510610, China; hnyuyang@stu.scau.edu.cn (L.L.); guoshuxuyujuan@163.com (Y.X.); ankejing2008@163.com (K.A.); 3 Institute of Agro-Products Processing, Yunnan Academy of Agricultural Sciences, Kunming 650032, China; sq@yaas.org.cn

**Keywords:** fermented mustard, biogenic amines, nitrite, microbial diversity

## Abstract

Biogenic amines (BAs) and nitrites are both considered harmful compounds for customer health, and are closely correlated with the microorganisms in fermented mustard (FM). In this study, BAs and nitrite contents in fifteen FM samples from different brands were analyzed. The concentrations of cadaverine in one sample and of histamine in one sample were above the toxic level. Moreover, five FM samples contained a high level of nitrite, exceeding the maximum residue limit (20 mg/kg) suggested by the National Food Safety Standard. Then, this study investigated bacterial and fungal communities by high-throughput sequencing analysis. *Firmicutes* and *Basidiomycota* were identified as the major bacteria and fungi phylum, respectively. The correlations among microorganisms, BAs and nitrite were analyzed. Typtamine showed a positive correlation with *Lactobacillus* and *Pseudomonas*. Cadaverine and nitrite is positively correlated with *Leuconostoc*. Furthermore, thirteen strains were selected from the samples to evaluate the accumulation and degradation properties of their BAs and nitrite. The results indicated that the *Lactobacillus* isolates, including *L. plantarum* GZ-2 and *L. brevis* SC-2, can significantly reduce BAs and nitrite in FM model experiments. This study not only assessed the contents of BAs and nitrite in FM samples, but also provided potential starter cultures for BAs and nitrite control in the FM products industry.

## 1. Introduction

Mustard, a unique vegetable in China, is distributed south of the Yangtze River. However, it is not ideal to be consumed as a fresh vegetable due to its bitter taste. Hence, mustard is spontaneously fermented as a kind of fermented vegetable. Fresh mustard is used as the main raw material to process fermented mustard (FM) by adding salt and other auxiliary materials. It is during this process that the mustard attaches itself to the microorganisms formed after fermentation [1]. Fermenting mustard not only gives it a more unique flavor, color and texture, but it also prolongs its storage period [2]. However, the quality of fermented vegetables highly depends on microorganisms; it is difficult to guarantee the safety of final fermented products because of the high amounts of harmful residues, such as BAs [3] and nitrite, which are frequently reported in fermented foods.

During fermentation, BAs are generally produced by microorganisms through enzymatic decarboxylation of amino acids and reductive amination of ketones and aldehydes [4]. Low doses of BAs can be quickly metabolised in the digestive tract [5,6], while a high level of BAs may produce some adverse consequences, such as headache, hot flushes and skin rashes [7]. In China, the content of BAs in fermented vegetables has no official limitations. Histamine is the only biogenic amine that is officially limited in fish Products, and it is regulated to be below 50 mg/kg by the US Food and Drug Administration and below 100 mg/kg by the European Community. Several studies have suggested maximum limits of BAs content in food products, i.e., 100 mg/kg for histamine, 100–800 mg/kg for tyramine, and 1000 mg/kg for total BAs content [8]. Lee et al. [9] reported that the concentrations of histamine and tyramine in fermented onions in Korea, exceeded recommended limits by a factor of four and two, respectively. Brink et al. [8] reported that histamine concentration in sauerkraut (a kind of fermented vegetable made with white cabbage in Europe) exceeded a recommended limit. However, BAs content in FM has not been evaluated in China until now.

Nitrite is a harmful compound and could cause many diseases [10,11]. Nitrite mainly comes from nitrate by the catalyzing of nitrate reductase, which is closely related to the microbial community [12]. Reduced iron (Fe^2+^) in haemoglobin can be oxidized by nitrite to its maximum oxidized state (Fe^3+^), thus reducing the total oxygen carrying capacity of the blood and causing methemoglobinemia. In addition, nitrite may be involved in the formation of *N*-nitrosamine by *N*-nitrosation reactions with dietary-derived amines in the stomach [13,14]. Liu, et al. [15] reported that the nitrite content in 65 out of 378 fermented vegetable samples was found to be above 20 mg/kg, which is the maximum nitrite content in fermented vegetables specified by the National Food Safety Standard. Therefore, it is necessary to control the formation of nitrite in FM.

There is a close correlation among BAs, nitrite and microorganisms in FM. This study investigated the contents of BAs and nitrite in FM from different brands, and found that the bacteria and fungi communities in FM were clarified by high-throughput sequencing analysis. In addition, strains highly capable of degrading nitrite and biogenic amines were screened from samples, and potential starter cultures for BAs and nitrite control in the FM products industry were provided.

## 2. Materials and Methods

### 2.1. Samples and Media

The top 15 fermented mustard samples from different brands were purchased according to the sales on Alibaba.com (Alibaba network technology co., Ltd., Hangzhou, China). Sample label uses a combination of regional and brand abbreviations. The information of all samples is shown in Table 1. All samples were packed well and purchased on Alibaba.com. Then they were stored at −20 °C for further analysis.

Certified analytical standards (putrescine, histamine, cadaverine, tryptamine, phenylethylamine, tyramine, spermidine ≥ 99%, spermine ≥ 99%, arginine, ornithine ≥ 99%, glutamine ≥ 99%, histidine, lysine, tryptophan ≥ 98%), 1,7-diaminoheptane assay 98% and dansyl chloride 97% were provided by Sigma-Aldrich (Darmstadt, Germany). Other analytical grade reagents were also purchased from Sigma-Aldrich.

### 2.2. Determination of Nitrite Concentration

**Figure 1:**
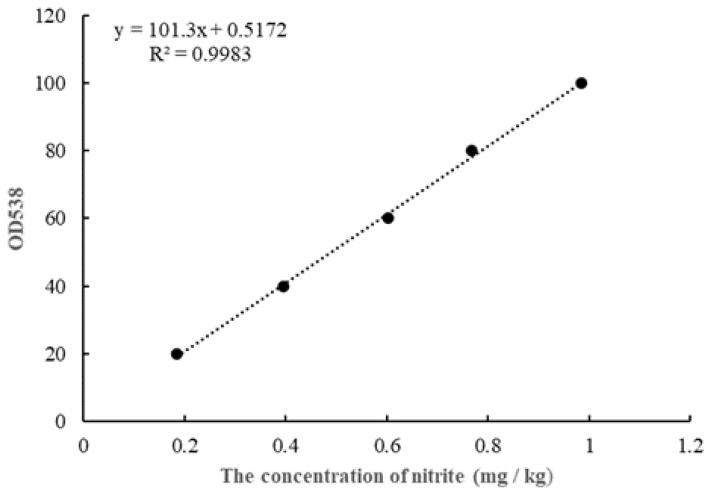
The standard curve for nitrite determination.

The nitrite concentration in FM samples was calculated by using the Griess reaction [16]. Briefly, an FM sample that weighs 4 g was crushed, deproteinated and defatted by 10 mL of 30% *w*/*v* ZnSO_4_·7H_2_O solution and 0.5 mL of 15% (*w*/*v*) K_4_Fe (CN)_6_·3H_2_O solution. Then, 1 mL of 0.2% sulfanilamide and 1 mL of 0.1% *N*-1-naphtyethylene diamine dihydrochloride were added sequentially to filtrates. This reaction required the room temperature and a duration of 15 min. The optical density (OD) of the colored mixtures was measured at 538nm against the reagent blank. The standard curve was obtained by performing the same color development process and OD analysis, which is shown in Figure 1.

### 2.3. Determination of BAs Concentrations

The methods described previously were used to measure the BAs content [17]. Briefly, 1 g of a sample was homogenized with 3 mL of 0.4 M HClO_4_ and extracted for 1 h. The mixture was centrifuged at 3000× *g* for 10 min, and the supernatant was collected. Subsequently, the supernatant (250 μL) was blended with 25 μL of NaOH (2 M) and 75 μL of saturated NaHCO_3_, and then reacted with 500 μL of dansyl-chloride (5 mg/mL) at 55 °C for 40 min. Later, the reactant was mixed with 25 μL of 25% NH_4_OH and incubated at 55 °C for 10 min. Then, a 0.22 μm membrane was utilized to filter the mixture for HPLC (high-performance liquid chromatography) analysis which was carried out by using an Agilent HPLC system with an Eclipse XDB-C18 (4.6 mm × 250 mm, 5 µm) column at 30 °C. The detection was analyzed at 254 nm. The elution solution was formed with a linear gradient of mobile phase A (acetonitrile) and B (H_2_O) at a flow rate of 1 mL/min. The solvent gradient was shown below: 0–4 min, 50% A; 4–22 min, 50–90% A; 22–30 min, 90–50% A; 30–35 min, 50% A. 

The applied analytical method was evaluated with limits of detection (LOD), and limits of quantification (LOQ), which were showed in Table 2. The LOD and LOQ of BAs ranged from 0.19–0.42 mg/kg and 0.53–1.27 mg/kg, respectively. The result suggested that those methods were suitable for BAs analyses.

### 2.4. Microbial Community Analysis

The E.Z.N.A Soil DNA kit (OMEGA, New York, NY, United States) was used to extract the genomic DNA from the FM samples. The V3-V4 region of the bacterial 16S rRNA genes was amplified with the primer 341F (5′-CCTACGGGNGGCWGCAG-3′) and 805R (5′-GACTACHVGGGTATCTAATCC-3′). The ITS1 region of fungal community was amplified with the primer ITS1F (5′-CTTGGTCATTTAGAGGAAGTAA-3′) and ITS2 (5′-GCTGCGTTCTTCATCGATGC-3′). After PCR and purification, a DNA library was built and operated on the Miseq Illumina platform at LC-Bio (Hangzhou, China).

Raw sequences generated by MiSeq sequencing and low-quality sequences were discarded using QIIME2. The UPARSE pipeline was used to analyze clean paired sequences retained for each sample and further generate operational tax-onomic units (OTUs) and screen representative sequences at 97% similarity.

### 2.5. Isolation and Purified of Lactic Acid Bacteria (LAB) from FM Samples

The mixtures of crushed FM samples (5 g) and 45 mL sterile saline were incubated (40 min; 35 °C). Then, samples were diluted (10^−1^ to 10^−6^) with sterile saline. As to each dilution, 200 µL sample solution was plated onto MRS agar plates and was incubated at 37 °C for 48 h. Then, observing and selecting colonies of medium size, raised, slightly white, moist, and the surrounding yellow, which was purified by spreading onto an MRS agar plate to obtain a single colony. The purified strains were initially identified as LAB *s* by Gram staining and hydrogen peroxide.

### 2.6. Strains Identification

Genomic DNA of bacteria was extracted using Tiangen Kit (Tiangen Biotech Co., Ltd., Beijing, China) and following the manufacturer’s protocol. The 16S rDNA sequence was amplified using the primers of 27f (AGAGTTTGATCMTGGCTCAG) and 1492r (CTACGGCTACCTTGTTA CGA). The PCR amplicons were sequenced and analyzed using the Blastn program.

### 2.7. Evaluation of BAs and Nitrite Production Ability

This study assessed the BAs production capability by culturing the strains in 5 mL MRS medium mixed with 1 g/L of histidine, tyrosine, tryptophan, phenylalanine, ornithine monohydrochloride, lysine or agmatine sulfate salt. Strains were also cultured in 5 mL MRS medium to measure the nitrite production capability. Finally, the production capability of BAs and nitrite was identified after incubating at 37 °C for 48 h.

### 2.8. Evaluation of BAs and Nitrite Degradation Ability

To measure the BAs and nitrite degrading ability, the cells were cultured and collected by centrifugation at 6000× *g* for 5 min. After washing with 0.05 mol/L phosphate buffer (pH = 7), the cell pellets were diluted to OD600 = 0.4 in phosphate buffer (0.05 mol/L) containing 100 mg/L of histamine, tyramine, tryptamine, β-phenethylamine, putrescine, cadaverine and nitrite cultured for 48 h to detect the residual BAs and nitrite. The blank phosphate buffer without cell pellets was used as the control. The inoculum density was 2% (*v*/*v*). The BA-degradation rate was measured based on the equation M = [(A − B)/A] × 100%, where M represents the BAs or nitrite degradation percentage and A and B indicate the original and final contents of BAs or nitrite, respectively [18].

### 2.9. Fermented Mustard Product Model Analysis

BAs and nitrite-controlling capacities of different strains were compared by using the fermented mustard model. Strain was inoculated into MRS broth at 37 °C for 12 h. The strain was then centrifuged at 4000 rpm for 10 min and washed three times with sterilized saline. The strain solution was dissolved with sterilized saline and adjusted to 1 × 10^8^ CFU/mL. The inoculum size was 5 mL strain solution/100 g cabbage, and the mustard mixed with equivalent sterile water was used as the control. After incubation for 15 days at 37 °C, the total contents of BAs and nitrite were measured.

## 3. Results and Discussion

### 3.1. BAs Contents in FM Samples

It was reported that putrescine, tyramine, cadaverine, histamine and tryptamine constitute a major part of BA profiles in sauerkraut [6,15]. This study found six common BAs with different contents in FM samples (Table 3), including tryptamine (11.02–35.74 mg/kg), putrescine (3.79–39.71 mg/kg), cadaverine (0–97.92 mg/kg), histamine (1.43–213.13 mg/kg), tyramine (0–37.85 mg/kg), and spermidine (0–3.39 mg/kg). The total content of BAs ranged from 34.57 to 295.86 mg/kg. Lee, et al. [9] studied BAs in 13 sauerkraut samples. The contents of BAs in sauerkraut ranged as follows: tryptamine, not detected (0–15.95 mg/kg); β-phenylethylamine, (0–5.97 mg/kg); putrescine, (0–254.47 mg/kg); cadaverine, (0–123.29 mg/kg); histamine, (8.67–386.03 mg/kg); tyramine, (0–181.10 mg/kg); spermidine, (2.32–18.74 mg/kg); spermine, (0–33.84 mg/kg). Mayr and Schieberle [19] studied BAs in sauerkraut and found putrescine (108.9 mg/kg), tyramine (60.66 mg/kg) and histamine (37.01 mg/kg) as major parts. Cadaverine and spermidine were identified at lower levels 21.5 and 10.98 mg/kg, respectively, while spermine (1.2 mg/kg) was at a very low level. There are differences in distribution and content of BAs in fermented vegetables from different countries, which may be caused by variations in manufacturing methods, major ingredients and storage conditions of FM samples used in the current and previous studies.

The total contents of BAs in fifteen FM samples were different. GZYY sample had the highest content of total BAs (259.86 mg/kg), especially tryptamine (35.74 mg/kg) and cadaverine (97.92 mg/kg). In contrast, SCYG sample showed the lowest concentration of total BAs (34.57 mg/kg), with cadaverine and spermidine not found. Excessive BAs will bring various harmful effects. Currently, there are no officially specified limits to BAs contents in fermented vegetables, whereas histamine is the only biogenic amine that is officially limited in fish products; it is regulated to be below 50 mg/kg by the US FDA [20]. However, several studies have suggested limits for BAs content in foods: 100 mg/kg for histamine, 100–800 mg/kg for tyramine and 1000 mg/kg for total content of BAs [9,21]. This study result indicated that the total content in all the samples was below the harmful level (1000 mg/kg), and samples were in the relative safe level on the whole. However, each BA has a different toxic level for humans. For example, histamine causes nausea, headache, hot flushes and skin rashes. Tyramine and tryptamine lead to migraine and hypertensive crises [22]. Furthermore, as shown in Table 3, 1 of 15 (6.67%) FM samples contained histamine whose level is higher than the limit (50 mg/kg) specified by the US FDA. Despite the existence of tryptamine and tyramine in most FM samples, their content was below the hazardous level (100 mg/kg). Peñas et al. [23] recommended maximum limits of 50 mg/kg and 25 mg/kg for putrescine and cadaverine, respectively. The concentration of putrescine in SCDS sample was over 25 mg/kg, and 8 of 15 (53.3%) samples had a higher level of cadaverine than this safe value, which might have a negative impact on human health. Thus, it is required to decrease the content of BAs in FM to improve safety.

### 3.2. Nitrite Contents in FM Samples

**Figure 2:**
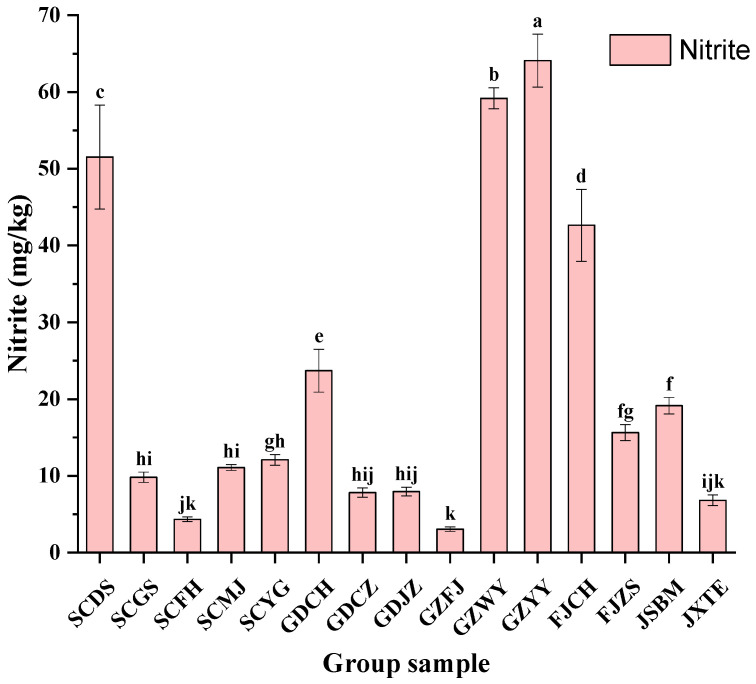
Contents of nitrite in FM samples. Different letters (a, b, c, etc.) indicate significantly different means at *p* < 0.05.

Figure 2 showed the concentrations of nitrite in 15 samples. GZYY sample had the highest nitrite content (64.09 mg/kg), followed by samples GZWY (59.18 mg/kg), SCDS (51.53 mg/kg) and FJCH (62.63 mg/kg). On the contrary, ZJHZ sample had the lowest nitrite concentration (32.24 mg/kg). Nitrite contents of different samples vary significantly. It was confirmed that the nitrite content increases as a result of microbial metabolism [24]. Certain strains of *Lactobacillus brevis*, *Lactobacillus plantarum* and *Leuconostoc. Mesenteroides* were found to be able to metabolize nitrite [25]. Furthermore, it was reported that enzymatic activity and microbial metabolism were influenced by some ingredients in fermented vegetables, such as salt, sugar, ginger and garlic, which could change the nitrite contents during the fermentation process [15].

According to National Food Safety Standard, the maximum limit for nitrite in fermented vegetables is 20 mg/kg. However, the nitrite contents in five FM samples (33.3%) exceeded the limit. Besides, the reaction between BAs and nitrite may produce nitrosamines with a well-known carcinogenic potential [26]. Therefore, controlling the content of BAs and nitrite is currently a top priority in China. The accumulation of BAs in fermented foods largely relies on the microorganisms that possess amino acids decarboxylases [27]. Similarly, the nitrate reductase catalyzing nitrite exists inside microorganisms [11]. Despite the great variation of BAs levels in fermented vegetables and the affection of vegetable varieties, fermentation conditions (pH, temperature, salt concentration, oxygen) and degree of microbiologic contamination, BAs and nitrite were significantly affected by complex microbial communities. Hence, evaluating the microbial communities and investigating their influence on BAs formation are especially important to control the BAs in FM samples.

### 3.3. Microbial Communities in FM Samples

As described above, contents of BAs and nitrite in FM samples vary largely, which probably results from diverse microbiological compositions. Thus, it is necessary to analyze the microbial communities in FM samples. By high-throughput sequencing, 1,236,107 16S rRNA (V3–V4 regions) and 1,258,811 ITS2 reads were generated from samples. After the quality control, 1,004,628 high-quality 16S rRNA gene sequences and 1,237,629 high-quality ITS gene sequences were obtained from each sample, with the average read length of 426 bp for bacteria and 211 bp for fungi. Alpha-diversity metrics (observed OTU, Chao1, Shannon and Good’s sample coverage) aim at evaluating richness and diversity of microbial communities (Table 4). Chao1 and Shannon indexes indicated microbial richness and diversity, respectively, throughout the samples. The Good’s coverage estimator was 99% for all samples, demonstrating that most bacterial and fungal phylotypes were found. SCYG sample had the maximum bacterial OTUs, and GDJZ sample had the highest fungal OTUs. Additionally, according to this study, the bacterial diversity was richer than that of fungi, manifesting that the bacteria were the most crucial microbial community in the fermentation process, which is consistent with previous reports [28].

**Figure 3:**
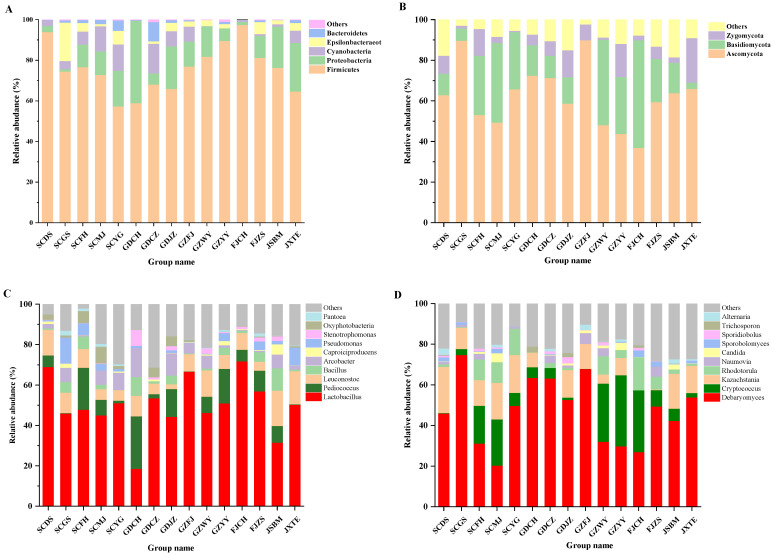
Microbial community in FM samples. (**A**)Bacteria at the phylum level, (**B**) Fungi at the phylum level, (**C**) Bacteria at the genus level, (**D**) Fungi at the genus level.

As to bacteria (Figure 3A), *Firmicutes* was a major phylum in all FM samples, especially in FJCH, with the relative abundance up to 97.43%. Observed in all samples, *Proteobacteria* were a second major phylum in GDCH, JXTE and GDJZ samples and accounted for 40.51%, 24.04%, and 21.03% of the total phylum, respectively, as shown in previous reports. This result was similar to a previous study by Liu [29], in which proteobacteria and Firmicutes were the dominant phylum during the period of mustard fermentation. The abundance of Firmicutes increased and became a major phylum as fermentation progressed, with conversely decreasing Proteobacteria.

At the genus level, *Lactobacillus* existed in all samples and was particularly enormous in FJCH, SCDS and GZFJ samples, accounting for up to 71.86%, 69.02% and 66.69% of total genus, respectively (Figure 3C). *Lactobacillus*, as the dominant lactic acid bacteria in fermented vegetables, could generate plenty of lactic acid to reduce the pH and enhance the acidity [29]. Besides, *Pediococcus*, *Leuconostoc* and *bacillus* were also widely distributed in FM samples, and are reported to be present in other traditionally fermented foods such as cheese, beer, wine, fermented soybeans [30] and Sichuan paocai [31]. Some strains of *Pediococcus* are reported to be able to grow under anaerobic conditions [32]. However, they were quite different from Korean kimchi whose dominant microbial communities are *Leuconostoc*, *Weissella* and *Lactobacillus* [33]. In fact, *Leuconostoc* and *Weissella* were not the main genera in the samples, possibly due to the influence of environmental factors, such as the concentration of salty water, fermentation period and temperature [15]. In addition, the genus *Arcobacter* was also highly abundant in some samples (GDCH, GDJZ, SCYG, etc.). Previous researchers have expressed that NaCl is beneficial to the growth of certain strains.

Figure 3B indicates the difference in fungal abundance in samples at phylum level. *Ascomycota* was the most abundant phylum in most FM samples, while *Basidiomycota* was the most popular phylum in FJCH sample. Both were reported to be the main fungi at phylum level in other types of traditionally fermented Chinese vegetables [32,34], which complies with this study. Figure 3D showed differences in fungal genera in various FM samples. *Debaryomyces* was the richest in most samples, probably due to its great tolerance to high concentration of NaCl and acid. *Cryptococcus* and *Kazachstania* were also the major fungi (≥20% in over one sample) in most samples. *Cryptococcus* was a ruling genus in samples GZYY (35.02%), FJCH (30.46%), GZWY (28.64%) and SCMJ (22.80%). *Kazachstania* was a prevailing genus in SCDS (22.80%) (Figure 3D). In addition, *Candida* and *Sporobolomyces* were found, even though they were reported to disappear rapidly during the later fermentation stage [35]. Both may include some opportunistic pathogenic strains and were usually related to many hospital-acquired infections [36]. Thus, consuming undercooked FM might have safety hazards.

*Arcobacter. Vibrio* has been found in salted cabbage or kimchi [37].

### 3.4. Correlations among Microbial Communities, Six Main BAs and Nitrite

**Figure 4:**
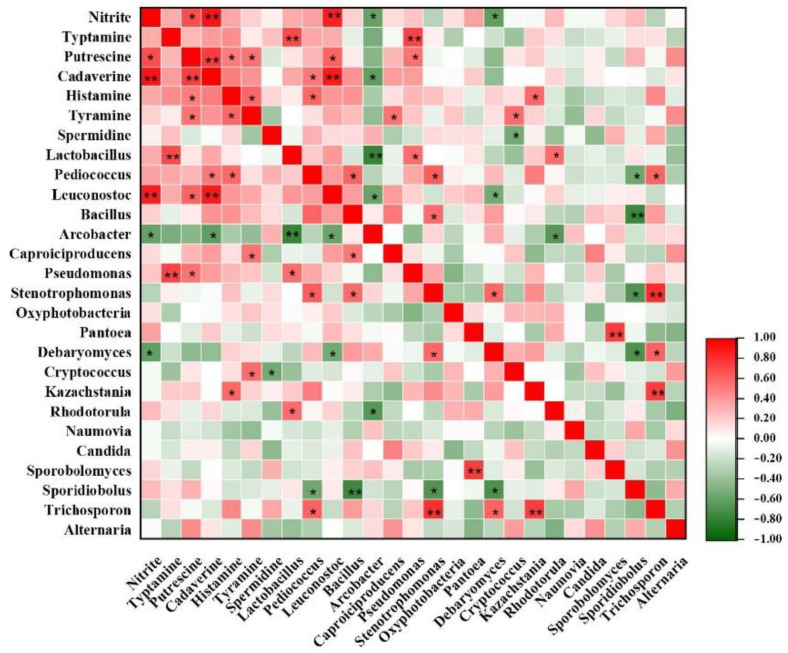
Heatmap of correlations among biogenic amines (BAs), nitrate microbial community and in FM samples. The red and green colors represent positive (0 < r < 1) and negative (−1 < r < 0) correlation, respectively. *, *p* < 0.05; **, *p* < 0.01.

Different microorganisms may produce varied amino acid decarboxylases which form different biogenic amines. Lactic acid bacteria (LAB) are the major microorganisms producing biogenic amines [38]. This paper studied the correlation between microbial communities and six main BAs in FM (Figure 4). The correlation between 10 genera and BAs in FM was positive, while the relation between another three genera and BAs was negative. Typtamine had a positive correlation (*p* < 0.001) with *Lactobacillus* (r = 0.72) *& Pseudomonas* (r = 0.72). Cadaverine and *Leuconostoc* (r = 0.89) showed the strongest positive correlation (*p* < 0.01). This result was similar to another two studies. Aflaki, F., et al. [39] found that *Lactobacillus*, *Pediococcus* and *Leuconostoc* are main producers of tryptamine or histamine in wine. Jin et al. found that typtamine and putrescine in Korean kimchi were produced mainly by *Lactobacillus* [40]. Besides, some new bacteria and fungi genera related to biogenic amines have been discovered, possibly resulting from the complicated production and accumulation of biogenic amines in fermented foods that are easily affected by various factors and their interactions.

Nitrite and *Leuconostoc* (r = 0.87) showed a positive correlation (*p* < 0.01) (Figure 4). A previous study found that certain strains of *Leuconostoc mesenteroides* obtained from kimchi were capable of metabolizing nitrite. In addition, strains of *Lactobacillus brevis* and *Lactobacillus plantarum* were reported to be able to metabolize nitrite during FM fermentation [41], but the correlation between nitrate and *Lactobacillus* has not been found in this study. This possibly results from the dissimilarities of fermentation temperature and NaCl concentration in different FM samples, which also affects the content of nitrite.

### 3.5. Microbial Contribution to BAs and Nitrite Contents in FM

In view of the results of microbial diversity in FM samples, the SCYG, GZFJ, JXTE, GDCZ, GDJZ, JXTE, SCFH and GZFJ FM samples were utilized to isolate strains. Lactic acid bacteria (LAB) are the most important strain in the fermentation process, which affects the quality (flavor, texture, nutrition) through metabolism [42,43]. In order not to affect the quality, we first screened *Lactobacillus* from fermented mustard. Thirteen strains initially identified as LAB by Gram staining and hydrogen peroxide, which were named SC-1, SC-2, SC-3, SC-4, SC-5, JX-1, JX-2, JX-3, GD-1, GD-2, GZ-1, GZ-2, and GZ-3.

To further explore the microbial contribution to BAs and nitrite contents in FM, this study evaluated the BAs production and degradation capabilities of these isolated strains. The BAs and nitrite production capabilities of all isolated strains were identified in medium, supplemented with corresponding precursors, and the BAs and nitrite generated by different strains are shown in Table 5. Some strains could produce histamine, tyramine, tryptamine, β-phenethylamine or cadaverine from corresponding substrates. Specifically, SC-4 produced the highest content of total BAs (77.71 mg/kg), especially a relatively high level of tyramine (30.54 mg/kg). In contrast, GZ-2 produced only putrescine via corresponding substrates, and generated the least content of total BAs (4.65 mg/kg). Most isolated strains (excluding SC-1, JX-3 and GZ-2) could produce nitrite. Furthermore, this paper studied the degrading capabilities of such thirteen isolates (Table 5). Some strains, including SC-4, JX-1, JX-2 and GZ-2, showed the degradation capability of BAs. Moreover, GZ-2 was highly capable of degrading nitrite.

### 3.6. The BAs and Nitrite Controlling Capacity of Selected Strains in FM Model

For the thirteen strains, GZ-2 and JX-3 showed a lower production capacity of BAs, but no nitrite producing ability (Table 5). SC-2 and GD-2 showed a high degradation rate of BAs and nitrite (Table 6). The 16S rDNA or ITS sequences were analyzed to identify these strains, and similarities of sequences of representative isolated strains are shown in Table 7. The result revealed that these strains were identified as *Lactobacillus plantarum* MLG5-1 (GZ-2); *Lactobacillus sp*. KLDS 1.0702 (JX-3); *Lactobacillus brevis* 6323 (SC-2); *Leuconostoc carnosum* JB16 (GD-2). Most of these strains are part of the dominant genera in FM.

**Figure 5:**
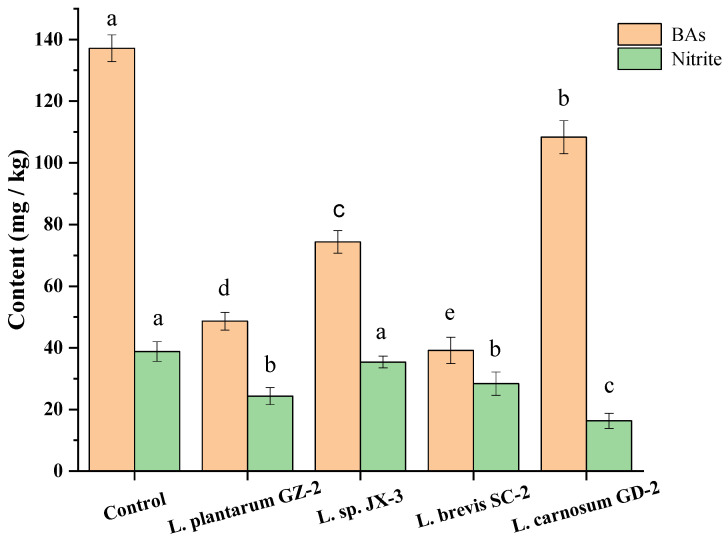
BAs and nitrite contents in model fermented mustard product inoculated with different strains. Different letters (a, b, c, etc.) indicate significantly different means at *p* < 0.05.

The above four strains were possibly useful candidates to control the BAs, and the *Lactobacillus* and *Leuconostoc* strains were commonly used as the starter cultures in fermented meat products [44]. Thus, their BAs-controlling properties were assessed in a FM model. After the fifteen-day fermentation, the total contents of BAs and nitrite in the control sample without inoculation achieved 137.16 mg/kg and 38.79 mg/kg (Figure 5). After inoculation with GZ-2, JX-3, SC-2 and GD-2, total concentrations of BAs and nitrite were much lower than those of the control group (Figure 5). At the same time, the lowest BAs content (39.16 mg/kg) was identified in the FM inoculated with *L.* *brevis* SC-2, and the lowest nitrite content (16.34 mg/kg) was found in the FM inoculated with *L. carnosum* GD-2. Among the four strains, *L. plantarum* GZ-2, *L. sp*. JX-3 and *L. brevis* SC-2 were weakly capable of forming BAs, while *L. plantarum* GD-2 exhibited a higher capability of forming BAs (108.34 mg/kg) (Figure 5). The *L. plantarum* GZ-2 and *L. carnosum* GD-2 with a lower capacity of BAs and nitrite production were more beneficial to decrease the contents of BAs and nitrite in FM products (Figure 5). Therefore, *L. plantarum* GZ-2 and *L. brevis* SC-2 were highly capable of controlling BAs and nitrite during FM processing. Xia et al. [41] reported that *Lactobacillus brevis* AR123 could significantly quicken degradation of nitrite and shorten the fermentation period for fermented vegetables. Xia et al. [15] reported that the concentrations of BAs and nitrite in pickled cabbage inoculated with starter cultures of *Lactobacillus plantarum* were remarkably lower than those in the spontaneous fermentation system during the entire fermentation process. In addition, Rabie et al. [45] reported that upon inoculation with *Lactobacillus plantarum* 2142 in fermented vegetables, the total biogenic amine contents remained considerably lower than those of the control. All the above results indicate that there are strains of *Lactobacillus plantarum* and *Lactobacillus brevis*, which can degrade biogenic amine or nitrite. However, the degradation of BAs and nitrite with *Leuconostoc carnosum* in fermented vegetables was reported.

## 4. Conclusions

In conclusion, the total BAs contents in all the fifteen FM samples were acceptable, while histamine and cadaverine were above the toxic level in some samples. Five samples contained nitrite with levels higher than the allowable limit (20 mg/kg). In addition, this paper studied bacterial and fungal communities by high-throughput sequencing analysis and identified correlations among microbes, BAs and nitrite. After the microbial community analysis, thirteen representative strains were chosen from the ruling microbial genera, and two *Lactobacillus* strains were identified to be beneficial to BAs control in the FM model. This study not only interpreted the microbial influence on BAs and nitrite accumulation in FM, but also provided potential starter cultures for BAs and nitrite control in the fermented meat products industry.

However, we recognize that this is a preliminary study. We only gained potential strains for BAs and nitrites control in FM products industry. In future, we will explore the intrinsic mechanisms by metagenomics and metabonomics, making clear the use of these strains for the reduction in BAs or nitrites in fermented mustard.

## Figures and Tables

**Table 1:** All sample information and labels in this study.

Number	Region	The Abbreviation of Brand Name	Label
1	Sichuan (SC)	DS	SCDS
2	Sichuan (SC)	GS	SCGS
3	Sichuan (SC)	FH	SCFH
4	Sichuan (SC)	MJ	SCMJ
5	Sichuan (SC)	YG	SCYG
6	Guangdong (GD)	CH	GDCH
7	Guangdong (GD)	CZ	GDCZ
8	Guangdong (GD)	JZ	GDJZ
9	Guizhou (GZ)	FJ	GZFJ
10	Guizhou (GZ)	WY	GZWY
11	Guizhou (GZ)	YY	GZYY
12	Fujian (FJ)	CH	FJCH
13	Fujian (FJ)	ZS	FJZS
14	Jiangsu (JS)	BM	JSBM
15	Jiangxi (JX)	TE	JXTE

**Table 2:** The limits of detection (LOD) and limits of quantification (LOQ) of analytical method for BAs.

Parameters	Putrescine	Cadaverine	Tyramine	Histamine	Tryptamine	Spermidine
LOD (mg/kg)	0.25	0.24	0.31	0.42	0.39	0.19
LOQ (mg/kg)	0.86	0.76	0.93	1.27	1.11	0.53

**Table 3:** Contents of BAs in FM samples. Data are presented as mean ±SDs of three replicates; ND means “not detected”. Different letters (a, b, c, etc.) indicate significantly different means at *p* < 0.05.

Group Sample.	Total BAs (mg/kg)	Tryptamine (mg/kg)	Putrescine (mg/kg)	Cadaverine (mg/kg)	Histamine (mg/kg)	Tyramine (mg/kg)	Spermidine (mg/kg)
GZYY	259.86 ± 6.23 ^a^	35.74 ± 1.34 ^a^	39.71 ± 3.95 ^b^	97.92 ± 2.33 ^a^	37.85 ± 1.66 ^b^	45.25 ± 0.46 ^a^	3.39 ± 0.19 ^a^
SCDS	199.45 ± 2.43 ^b^	13.75 ± 1.49 ^g,h^	61.75 ± 1.35 ^a^	62.80 ± 0.46 ^d^	31.35 ± 0.31 ^c^	29.79 ± 1.42 ^e^	ND
SCFH	195.92 ± 3.5 ^b,c^	14.60 ± 1.22 ^f,g,h^	24.72 ± 1.27 ^d^	81.89 ± 0.18 ^b^	24.55 ± 1.1 ^f^	47.64 ± 1.21 ^a^	2.52 ± 0.06 ^a,b^
FJZS	189.50 ± 0.74 ^c,b^	17.59 ± 0.04 ^e^	31.60 ± 0.12 ^c^	74.68 ± 1.53 ^c^	27.22 ± 0.82 ^d,e^	36.11 ± 1.26 ^c^	2.30 ± 0.02 ^a,b^
JSBM	184.90 ± 3.47 ^c,d^	11.76 ± 0.08 ^i,j^	27.50 ± 0.52 ^d^	75.16 ± 0.06 ^c^	29.61 ± 1.77 ^c,d^	40.88 ± 1.32 ^b^	ND
FJCH	157.17 ± 4.68 ^e^	26.76 ± 0.61 ^b^	26.14 ± 1.76 ^d^	49.11 ± 1.7 ^e^	25.09 ± 1.47 ^f^	37.03 ± 1.13 ^e,f^	2.54 ± 0.13 ^a,b^
GDJZ	137.70 ± 1.13 ^f^	20.45 ± 0.89 ^d^	18.02 ± 0.93 ^e,f^	10.57 ± 0.34 ^h^	62.05 ± 0.88^a^	23.35 ± 0.15 ^g^	3.27 ± 0.02 ^a^
SCMJ	125.88 ± 3.58 ^g^	14.28 ± 0.26 ^f,g,h^	15.74 ± 0.51 ^f,g^	41.2 ± 1.17 ^f^	19.93 ± 0.62 ^g^	32.30 ± 0.93^d^	ND
GDCH	102.84 ± 4.84 ^h^	15.73 ± 0.72 ^f^	14.72 ± 1.2 ^f,g^	26.5 ± 0.68 ^g^	25.29 ± 0.67 ^e,f^	17.47 ± 1.43 ^i^	3.13 ± 0.15 ^a^
GZWY	83.22 ± 3.85 ^i^	24.80 ± 0.29 ^c^	13.65 ± 1.64^fg^	11.97 ± 0.60 ^h^	12.61 ± 0.9 ^h^	20.18 ± 0.41 ^h^	ND
SCGS	72.83 ± 1.72 ^j^	13.89 ± 0.64 ^g,h^	8.21 ± 1.3 ^h^	9.68 ± 0.4 ^h^	11.62 ± 1.08 ^h^	26.4 ± 0.78 ^f^	3.03 ± 0.13 ^a^
GDCZ	69.45 ± 4.04 ^j^	10.34 ± 0.89 ^j^	19.32 ± 1.17 ^e^	6.54 ± 0.24 ^i^	ND	29.87 ± 1.93 ^e^	3.38 ± 1.59 ^a^
JXTE	38.91 ± 2.15 ^k^	14.89 ± 0.21 ^f,g^	4.58 ± 1.34 ^i^	ND	5.18 ± 0.14 ^i^	12.88 ± 2.07 ^j^	2.39 ± 0.22 ^a,b^
GZFJ	35.40 ± 3.23 ^k^	11.02 ± 0.22 ^i,j^	4.99 ± 0.76 ^i^	ND	ND	16.68 ± 1.42^i^	1.96 ± 0.83 ^b^
SCYG	34.57 ± 4.50 ^k^	12.85 ± 0.14 ^h,i^	3.79 ± 0.98 ^i^	ND	4.90 ± 1.96 ^i^	13.03 ± 1.7 ^i^	ND

**Table 4:** Alpha diversity metrics of microbial community in FM samples.

Samples	Bacteria				Fungi			
	Observed OUTs	Chao1	Shannon	Goods Coverage (%)	Observed OUTs	Chao1	Shannon	Goods Coverage (%)
GZYY	282 ± 12	283 ± 19	5.43 ± 0.34	99	22 ± 2	0.04 ± 0	23 ± 3	99
SCDS	258 ± 14	261 ± 35	5.77 ± 0.14	99	15 ± 1	0.03 ± 0	18 ± 2	99
SCFH	349 ± 36	348 ± 44	5.03 ± 0.23	99	27 ± 3	0.27 ± 0.01	25 ± 4	99
FJZS	337 ± 10	342 ± 28	4.78 ± 0.16	99	32 ± 3	0.3 ± 0.02	32 ± 5	99
JSBM	302 ± 16	303 ± 26	5.67 ± 0.28	99	14 ± 1	0.02 ± 0	14 ± 3	99
FJCH	170 ± 8	170 ± 13	4.51 ± 0.24	99	13 ± 4	0.04 ± 0	15 ± 4	99
GDJZ	312 ± 23	314 ± 19	4.94 ± 0.26	99	60 ± 6	0.57 ± 0.04	64 ± 7	99
SCMJ	299 ± 14	307 ± 27	5.63 ± 0.46	99	19 ± 2	0.04 ± 0	19 ± 4	99
GDCH	292 ± 8	298 ± 40	5.74 ± 0.2	99	29 ± 3	0.44 ± 0.13	29 ± 5	99
GZWY	186 ± 6	188 ± 16	4.55 ± 0.16	99	17 ± 6	0.04 ± 0	19 ± 4	99
SCGS	421 ± 15	436 ± 37	5.46 ± 0.26	99	26 ± 3	0.29 ± 0.01	25 ± 3	99
GDCZ	404 ± 31	408 ± 35	5.33 ± 0.18	99	30 ± 2	0.22 ± 0.03	32 ± 5	99
JXTE	390 ± 22	394 ± 46	5.15 ± 0.45	99	37 ± 4	0.34 ± 0.04	40 ± 6	99
GZFJ	187 ± 22	187 ±1 8	5.40 ± 0.23	99	22 ± 5	0.04 ± 0	21 ± 3	99
SCYG	436 ± 28	408 ± 51	5.49 ± 0.35	99	25 ± 3	0.29 ± 0.02	25 ± 2	99

**Table 5:** The BA-producing abilities of the isolated strains with corresponding precursor. Different letters (a, b, c, etc.) indicate significantly different means at *p* < 0.05.

Strain	Tryptamine (Tryptophan) (mg/kg)	Putrescine (Agmatine Sulfate Salt) (mg/kg)	Cadaverine (Lysine) (mg/kg)	Histamine (Histidin) (mg/kg)	Tyramine (Tyrosine) (mg/kg)	Total BAs (mg/kg)	Nitrite (mg/kg)
SC-1	7.54 ± 0.93 ^c^	13.45 ± 1.14 ^c^	ND	ND	26.31 ± 1.05 ^b^	47.3 ± 1.67 ^c^	ND
SC-2	ND	ND	ND	8.53 ± 0.76 ^b^	5.42 ± 0.68 ^g^	13.95 ± 0.96 ^h^	39.52 ± 1.87 ^c^
SC-3	ND	5.34 ± 0.93 ^e^	10.65 ± 0.57 ^b^	ND	20.31 ± 1.34 ^c^	36.30 ± 1.08 ^d^	53.31 ± 3.34 ^a^
SC-4	18.24 ± 1.07 ^a^	ND	5.42 ± 0.37 ^c^	23.51 ± 1.24 ^a^	30.54 ± 1.73 ^a^	77.71 ± 1.73 ^a^	8.62 ± 1.26 ^f^
SC-5	12.43 ± 1.31 ^b^	10.24 ± 1.05 ^d^	17.61 ± 1.64 ^a^	ND	6.31 ± 0.61 ^g^	56.59 ± 1.15 ^b^	36.42 ± 1.71 ^c^
JX-1	10.34 ± 0.92 ^bc^	4.65 ± 0.61 ^e^	ND	ND	14.65 ± 0.91 ^d^	29.64 ± 0.83 ^e^	16.34 ± 1.93 ^e^
JX-2	ND	17.15 ± 0.98 ^b^	ND	10.32 ± 0.37 ^b^	ND	27.47 ± 0.79 ^f^	25.86 ± 2.76 ^d^
JX-3	5.85 ± 0.43 ^d^	ND	ND	ND	7.12 ± 0.61 ^f,g^	12.97 ± 0.92 ^h^	ND
GD-1	4.50 ± 0.82 ^d,e^	ND	ND	ND	8.62 ± 0.34 ^e^	13.12 ± 0.71 ^h^	37.65 ± 2.08 ^c^
GD-2	7.45 ± 0.34 ^c^	23.65 ± 1.62 ^a^	9.61 ± 0.83 ^b^	ND	15.43 ± 0.57 ^d^	56.14 ± 1.01 ^b^	4.86 ± 1.17 ^g^
GZ-1	3.43 ± 0.72 ^e^	9.15 ± 0.93 ^d^	ND	ND	7.61 ± 0.81 ^f^	20.19 ± 0.54 ^g^	46.17 ± 2.43 ^b^
GZ-2	ND	4.65 ± 0.76 ^e^	ND	ND	ND	4.65 ± 0.49 ^i^	NG
GZ-3	8.64 ± 0.87 ^c^	16.54 ± 1.21 ^b^	ND	ND	9.31 ± 0.69 ^e^	34.49 ± 1.73d	16.23 ± 1.72 ^e^

**Table 6:** The BA-degradation abilities of the isolated strains with corresponding precursor. Different letters (a, b, c, etc.) indicate significantly different means at *p* < 0.05.

Strain	Tryptamine (%)	Putrescine (%)	Cadaverine (%)	Histamine (%)	Tyramine (%)	Nitrite (%)
SC-1	0	8.32 ± 1.14 ^c^	0	0	17.32 ± 2.16 ^a^	44.26 ± 2.34 ^a^
SC-2	0	0	0	0	0	13.46 ± 3.46 ^d^
SC-3	0	0	0	0	0	0
SC-4	10.32 ± 1.24 ^c^	0	17.32 ± 1.68 ^a^	23.65 ^a^	0	14.62 ± 2.17 ^d^
SC-5	0	0	0	0	0	0
JX-1	15.36 ± 0.94 ^a^	0	0	0	0	6.32 ± 1.92 ^e^
JX-2	0	21.35 ± 2.16 ^a^	0	0	0	0
JX-3	0	0	0	0	0	3.26 ± 1.14 ^f^
GD-1	0	0	0	0	0	0
GD-2	0	0	10.64 ± 0.92 ^b^	0	9.56 ± 1.16 ^b^	28.32 ± 2.09 ^b^
GZ-1	0	0	0	0	0	4.56 ± 1.21 ^e,f^
GZ-2	18.62 ± 1.86 ^b^	16.35 ± 1.34 ^b^	0	0	0	21.62 ± 3.42 ^c^
GZ-3	0	0	0	0	0	0
SC-1	0	0	0	0	0	38.41 ± 3.91 ^a^
SC-2	0	0	0	0	0	7.32 ± 1.73 ^e^

**Table 7:** 16S rDNA sequences similarities of isolated strains with representative microbe.

Isolates	Closest Strains	Identities (%)	Accession No.
GZ-2	*Lactobacillus plantarum* MLG5-1	99	EU600906.1
JX-3	*Lactobacillus plantarum.* KLDS 1.0702	99	MT473388.1
SC-2	*Lactobacillus brevis* ATCC 14869	99	NR044704.2
GD-2	*Leuconostoc carnosum* JB16	99	HV538100.1

## Data Availability

The data presented in this study is contained within this article.

## Sample Availability

Samples of the fermented mustard in this study are available from the authors.
